# Detection of Japanese Encephalitis Virus Genotype V in *Culex orientalis* and *Culex pipiens* (Diptera: Culicidae) in Korea

**DOI:** 10.1371/journal.pone.0116547

**Published:** 2015-02-06

**Authors:** Hyunwoo Kim, Go-Woon Cha, Young Eui Jeong, Wook-Gyo Lee, Kyu Sik Chang, Jong Yul Roh, Sung Chan Yang, Mi Yeoun Park, Chan Park, E-Hyun Shin

**Affiliations:** 1 Division of Medical Entomology, Center for Immunology and Pathology, Korea National Institute of Health, Cheongju, South Korea; 2 Division of Arboviruses, Center for Immunology and Pathology, Korea National Institute of Health, Cheongju, South Korea; 3 Department of Agricultural Biotechnology, Seoul National University, Seoul, South Korea; 4 Department of Biomedical Sciences, Graduate School of Hallym University, Chuncheon, South Korea; University of Liverpool, UNITED KINGDOM

## Abstract

Japanese encephalitis virus (JEV) causes significant viral encephalitis and is distributed throughout the Asian countries. The virus is known to be transmitted by *Culex tritaeniorhynchus*, which mainly breeds in rice paddies in Korea. In this study, we investigated the presence of other mosquito species that can transmit JEV as a second or regional vector. We selected five cities where patients have experienced JE in the last 5 years as mosquito-collecting locations and subdivided them into four collection sites according to the mosquito habitats (cowshed, downtown area, forest, and swamp). Mosquitoes were caught using the BG-Sentinel trap, CDC black-light trap, Fay-Prince trap, and Gravid trap. A total of 993 pools from 22,774 mosquitoes were prepared according to their species, collection date, and site. We performed a SYBR Green 1-based real-time RT-PCR assay to detect JEV from the mosquito pools. A total of six JEV-positive pools were detected from *Culex orientalis* and *Culex pipiens* caught in the Gangwon-do and Gyeonngi-do provinces. All the detected JEVs were revealed as genotype V by phylogenetic analysis of the envelope gene. Our findings confirm that a new genotype of JEV was introduced in Korea and suggest that two mosquito species may play a role in JEV transmission.

## Introduction

Japanese encephalitis virus (JEV) is a mosquito-borne RNA virus in the genus *Flavivirus* (family Flaviviridae) and causes approximately 30,000–50,000 human encephalitis cases each year throughout Asian countries [1,2]. JEV is of a circulating nature, forming a transmission cycle from mosquitoes to birds and swine, which together form the virus’s reservoir, and the swine act as an amplifying host. Thereafter, the cycle continues to mosquitoes, and then to humans or some incidentally infected vertebrates. JEV has five genotypes (I–V) based on the genetic distances of the envelope gene or complete genome sequences when it has only one serotype [2].

JEV is distributed in temperate and tropical areas of eastern and southern Asia, extending to India and Pakistan in the west. In these areas, rice irrigation is a common agricultural method. This ecosystem provides a good habitat for paddy-breeding mosquitoes such as *Culex tritaeniorhynchus*, the major vector mosquito of JEV in most parts of Asia, and other *Culex* mosquitoes that play roles as secondary or regional vectors [3]. Since these species share a similar ecological niche in irrigated rice paddies, JE is largely associated with rural areas [4]. In South Korea, JE has been well controlled after vaccine importation in the late 1970s. An extensive surveillance program, the JE epidemic forecast program, has been conducted since 1975 [5]. Through this program, vector mosquito density was monitored on a weekly basis, and the JEV was isolated from the mosquitoes. Antibody levels in unvaccinated pigs were also monitored in order to predict an epidemic. As a result, annual JE cases have been below ten after the last epidemic in 1983 (139 cases). We experienced an abrupt increase in JE cases in 2010, with five out of 26 cases occurring in Gangwon-do where *Culex trtitaneniorhyncus* mosquitoes are rarely distributed during the JE season (August to October) [6]. While health authorities investigated the reason for this increase based on mosquito density and antibody levels in pigs, they could not provide a clear explanation for the JE outbreak in an unexpected province. A possible answer was given in an article published by a US military research group in South Korea [7]. They detected JEV genotype V from *Culex bitaeniorhynchus* mosquitoes in north Gyeonggi-do in 2010, which was the third case following the previous reports in Malaysia and China [8,9]. In Taiwan, Chen et al suggested that the detection of a JEV antibody on a rice-free islet might be related to another potential vector mosquito found in similar ecological conditions [4]. These data indicate that JEV is rampant in nature and suggest the possibility of new vector-mosquito involvements in the natural cycle of JEV.

We also reviewed the current mosquito trapping methods and collection sites. In the JE epidemic forecast program, black-light traps were mainly used to capture mosquitoes, and the collection sites are primarily near cowsheds in villages. This condition would be acceptable for the purpose of the program (to detect JEV activity near human habitats and to take timely preventive measures) but does not reflect the distribution of mosquito species in various habitats. This makes it difficult to investigate the virus activity in nature. In this study, we investigated the presence of new mosquito species that may transmit JEV in a variety of different habitats, and using various mosquito traps.

## Materials and Methods

### Ethics Statement

The animal protocol used in this study was reviewed and approved based on its ethical procedures and scientific care by the KCDC-Institutional Animal Care and Use Committee (KCDC-IACUC).

### Mosquito collection

**Figure 1:**
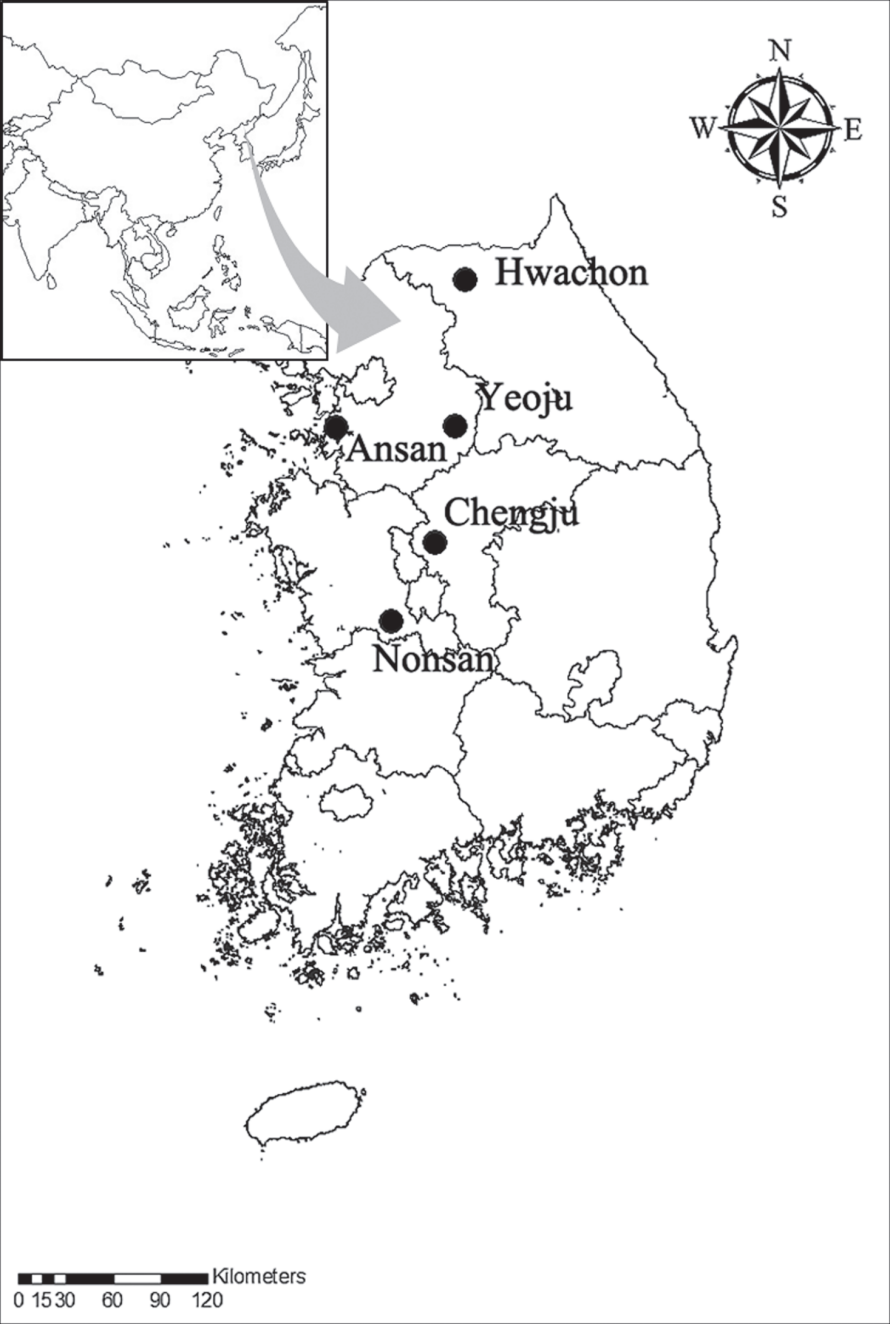
Locations of mosquito collection in South Korea. Mosquitoes were caught in five cities (four sites per city) once a month from May through October 2012.

Mosquitoes were collected from May through October 2012 in five cities (Ansan, Cheongju, Hwacheon, Nonsan, and Yeoju) where JE patients occurred from 2007 to 2011 in South Korea (Fig. 1). Each city was subdivided into four collecting sites (cowshed, downtown area, forest, and swamp). Four different types of traps: A CDC black-light trap (John W. Hock, USA), BG-Sentinel trap (Biogents AG, Germany), Fay-Prince trap (John W. Hock, USA), and CDC-Gravid trap (John W. Hock, USA) were placed in each habitat and operated once a month from 4:00 pm to 10:00 am. Trap indices (TI: mean number of female mosquitoes collected per trap per night) were determined for each site. There was no need for specific permission for using these collecting sites, because these sites were not located at national parks or protected areas, and mosquito collecting was supported by each local Public Health Center. Collected mosquitoes were killed by freezing and stored in an icebox containing dry ice. They were then transported to the laboratory.

### Virus detection

Female mosquitoes were identified morphologically and labeled based on the date and geographic region of their capture. Each mosquito pool (up to 50 mosquitoes) was placed in a 2-ml sterile tube containing four to six 2.5-mm-diameter glass beads. Different amounts of sterile phosphate-buffered saline (PBS) were then added to each tube according to the number of mosquitoes contained therein (0.3 ml PBS for up to 10 mosquitoes, 0.6 ml PBS for 11–20 mosquitoes, and 1.2 ml PBS for 21–50 mosquitoes). Mosquitoes were homogenized using automatic equipment, FastPrep (MP Biomedicals, Solon, OH, USA), 2 times for 20 s at 5000 rpm. Homogenates were placed on ice for at least 5 min, clarified by brief centrifugation, and used for RNA extraction.

The viral RNAs were extracted from the clarified supernatant of the homogenates using a QIAamp viral RNA mini kit (QIAGEN, Hilden, Germany) according to the manufacturer’s instructions. The RNAs were analyzed using one-step SYBR Green 1-based real-time RT-PCR with flavivirus group-specific primers detecting the NS 5 partial gene [10]. Verso SYBR Green 1-Step QRT-PCR ROX (Thermo, Waltham, MA, USA) and StepOnePlus instruments (Applied Biosystems, Foster City, CA, USA) were used for the RT-PCR assay. The reaction mixture (25 μl) contained 12.5 μl of 1-Step QRT-PCR SYBR ROX mix, 1.75 μl of each of the three primers (FL-F1, FL-R3, and FL-R4, 1μM stock), 1.25 μl of RT enhancer, 0.25 μl of verso enzyme, 0.75 μl of RNase/DNase free water, and 5 μl of the RNA sample. The thermal cycling consisted of reverse transcription at 50°C for 30 min, activation of *Taq* polymerase at 95°C for 15 min followed by 45 cycles of PCR (94°C for 15 s, 58°C for 20 s, 72°C for 30 s). After amplification, a melting-curve analysis was performed to verify each product by its specific melting temperature. Positive reactions, including for dengue virus, Japanese encephalitis virus, yellow fever virus, and West Nile virus showed melting temperatures ranging from 81°C to 85°C. The expected size of the positive product was approximately 212 bp in agarose gel electrophoresis.

The positive products were extracted from the gels using the QIAquick gel extraction kit (QIAGEN) and sequenced with primers FL-F1, FL-R3, and FL-R4. PCR direct sequencing was done in both directions using the ABI PRISM BigDye Terminator Cycle Sequencing kits and an ABI 3730xl sequencer (Applied Biosystems) at Macrogen (Seoul, South Korea). The resulting nucleotide sequences were done via an NCBI-BLAST search to identify the exact pathogens involved.

### Nucleotide sequencing and phylogenetic analysis

We designed five set of primer to obtain the complete envelope gene for JEV-positive pools (Table 1). The primers were designed manually by comparing two sequences of JEV genotype V (XZ0934 and 10–1827) available from GenBank. The eight microliters of RNA extracted from mosquito homogenate were reverse transcribed with a random hexamer using the SuperScript III First-Strand Synthesis System (Invitrogen, CA, USA) according to the manufacturer’s protocol. Then, synthesized cDNA was amplified with each primer set using the DyNAzyme EXT DNA Polymerase. The composition of reaction mixture was 5 μl of 10× optimized buffer, 1 μl of dNTP mix, 2.5 μl of each of the forward and reverse primers (10 μM stock), 1 μl of DNA Polymerase, 36 μl of RNase/DNase free water, and 2 μl of the cDNA template. The thermal profile for the PCR was as follows: pre-denaturation at 94°C for 2 min, 40 cycles of 94°C for 30 s, 60°C for 20 s (55°C for primer set of 1240F and 1734R), 72°C for 20 s, and a final extension cycle at 72°C for 3 min. The amplified fragments were visualized in agarose gel and extracted for nucleotide sequencing. PCR direct sequencing was done at Macrogen (Seoul, South Korea) as described in the section above. The resulting sequence files were compiled using the SeqMan program in the Lasergene software version 8.0 (DNASTAR, WI, USA) and the final 1,500 bp of the envelope gene sequences were obtained. The nucleotide sequences were compared with those of 38 other JEV strains deposited in the GenBank (Table 2). The phylogenetic analyses were performed using MEGA 6.06 [11]. A multiple alignment was generated with the MUSCLE program [12] in MEGA and the best evolutionary model for estimating genetic distances between sequences was estimated using the ‘Models’ function in MEGA. Phylogenetic trees were inferred by both distance- and character-based methods. For the distance method, a Neighbor Joining (NJ) tree was constructed using the evolutionary model of Tamura-Nei and the rate variation among sites was estimated from a gamma distribution (shape parameter = 5). The reliability of the tree was tested by bootstrap methods with 1,000 replications. For the character-base method, a Maximum Likelihood (ML) tree was constructed using the same substitution model and gamma distribution. The envelope gene sequence of the West Nile virus was used as the out-group (B956strain, GenBank accession number: NC_001563). Detailed parameters for the tree construction are available upon request from the corresponding author.

**Table 1:** Primers for amplification of the complete envelope gene of JEV genotype V.

**Primer**	**Sequence (5′-3′)**	**Position^†^**	**Polarity**	**Size (bp)**
69F	TGCAAACCCACGGAGAA	763–779	Forward	501
550R	GCCTTGCTTGCAGACATAG	1262-1244	Reverse	
373F	CCTCACTATCATGGCGAACGACA	1067–1089	Forward	557
908R	CGCGATGGACTAGGAACGACTTA	1623-1601	Reverse	
819F	AGCTTGGAGATTACGGAGAGGTCA	1513–1536	Forward	572
1370R	CTTCGAATTGGCGGTGGATGT	2084-2064	Reverse	
1240F	TGGTACGGTTGTCATAGAA	1934–1952	Forward	512
1734R	CACCTCCTGTAGCAAGAA	2445-2428	Reverse	
1622F	GGAGCTTTCAGAACCCTTTTTG	2316–2337	Forward	378
1980R	CCTGACGGCTTCCCACATTT	2693-2674	Reverse	

^†^ The nucleotide position was based on the JEV XZ0934 strain (Genotype V, GenBank accession number, JF915894).

**Table 2:** Details of the Japanese encephalitis viruses used in the phylogenetic analysis.

**Strain**	**Genotype**	**Location**	**Sampling year**	**GenBank accession number.**
YN86-B8639	**1**	China	1986	DQ404133
SH80	**1**	China	2001	JN381848
3XG009	**1**	China	2011	JX514950
IND/11/WB/JEV45	**1**	India	2011	KC526872
99P104	**1**	Japan	1999	FJ943474
09P123	**1**	Japan	2009	GU108334
K94P05	**1**	South Korea	1994	U34929
K01-JN	**1**	South Korea	2001	FJ938222
K05-GS	**1**	South Korea	2005	FJ938223
A10.825	**1**	South Korea	2010	JN587259
K10CT661	**1**	South Korea	2010	JX018150
TC2009-11	**1**	Taiwan	2009	JF499801
CY2010-3	**1**	Taiwan	2010	JF499824
ThCMAr4492	**1**	Thailand	1992	D45362
JE_CM_1196	**1**	Thailand	2005	DQ238602
90VN70	**1**	Vietnam	1990	HM228921
VN88	**1**	Vietnam	2001	AY376464
07VN310	**1**	Vietnam	2007	HM228922
FU	**2**	Australia	1995	AF217620
JKT5441	**2**	Indonesia	1980-Jun	JQ429306
BN19	**3**	China	1982	FJ185038
YN03-A151	**3**	China	1998	DQ404136
SCDJY01	**3**	China	2011	JX045833
GP78	**3**	India	1978	AF075723
IND/12/WB/JEV50	**3**	India	2012	KC526871
JaOArS982	**3**	Japan	1982	M18370
JaNAr0290	**3**	Japan	1990	AY427794
K88A071	**3**	South Korea	1988	FJ938228
K94A071	**3**	South Korea	1994	FJ938217
CH1392	**3**	Taiwan	1990	AF254452
YL0506a	**3**	Taiwan	2005	GQ260611
HL0805a	**3**	Taiwan	2008	GQ260628
VN207	**3**	Vietnam	1986	AY376461
04VN75	**3**	Vietnam	2004	HQ009263
JKT6468	**4**	Indonesia	1981	AY184212
XZ0934	**5**	China	2009	JF915894
Muar	**5**	Malaysia	1952	HM596272
10-1827	**5**	South Korea	2010	JN587258

## Results

### Mosquito collection

A total of 20,774 mosquitoes representing 9 genera and 20 species were collected, including 9 species of *Culex*, 2 species of *Aedes*, 3 species of *Ochlerotatus*, and *Armigeres, Coquillettidia, Culiseta, Mansonia, Tripteroides*, and *Anopheles* complex. The predominant species were *Cx. pipiens* (44.7%) and *Ae. vexans* (18.0%). Details are presented in Table 3. The TI of all traps (Table 4) during the study period was 45.5, ranging from a low of 0.9 (Hwacheon, at GV) to a high of 337.5 (Ansan, at BG) female mosquitoes per trap night.

**Table 3:** Total number mosquitoes collected at 5 cities in South Korea.

**Species**	**Cheongju (%)**		**Nonsan (%)**		**Ansan (%)**		**Yeoju (%)**		**Hwacheon (%)**		**Total (%)**	
***Culex bitaeniorhynchus***	6	(0.1)	2	(0.1)	34	(0.4)	8	(0.4)			50	(0.2)
***Culex hayshii***							3	(0.1)	1	(0.1)	4	(<0.1)
***Culex inatomii***			2	(0.1)	468	(4.9)					470	(2.3)
***Culex mimeticus***			1	(<0.1)							1	(<0.1)
***Culex orientalis***	83	(2.1)	73	(2.3)	31	(0.3)	264	(12.0)	47	(2.6)	498	(2.4)
***Culex pipiens***	1,155	(28.7)	757	(23.8)	6,254	(65.5)	917	(41.5)	208	(11.5)	9,291	(44.7)
***Culex rubensis***	1	(<0.1)									1	(<0.1)
***Culex tritaeniorhyncus***	2	(<0.1)	3	(0.1)	1	(<0.1)	4	(0.2)			10	(<0.1)
***Culex vagans***	3	(0.1)			2	(<0.1)					5	(<0.1)
***Aedes albopictus***	110	(2.7)	180	(5.7)	191	(2.0)	73	(3.3)	9	(0.5)	563	(2.7)
***Aedes vexans***	1,135	(28.2)	928	(29.1)	164	(1.7)	602	(27.3)	917	(50.8)	3,746	(18.0)
***Ochlerotatus dorsalis***					6	(0.1)					6	(<0.1)
***Ochlerotatus koreicus***	544	(13.5)	5	(0.2)	63	(0.7)	7	(0.3)	7	(0.4)	626	(3.0)
***Ochlerotatus nipponicus***									2	(0.1)	2	(<0.1)
***Anopheles spp.*^†^**	353	(8.8)	846	(26.6)	84	(0.9)	210	(9.5)	552	(30.6)	2,045	(9.8)
***Armigeres subalbatus***	551	(13.7)	373	(11.7)	31	(0.3)	117	(5.3)	60	(3.3)	1,132	(5.4)
***Coquillettidia ochracea***	11	(0.3)			104	(1.1)					115	(0.6)
***Culiseta bergrothi***					1	(<0.1)					1	(<0.1)
***Mansonia uniformis***	59	(1.5)			2,116	(22.2)	1	(<0.1)			2,176	(10.5)
***Tripteroides bambusa***	15	(0.4)	14	(0.4)			2	(0.1)	1	(0.1)	32	(0.2)
**Total**	4,028	(100.0)	3,184	(100.0)	9,550	(100.0)	2,208	(100.0)	1,804	(100.0)	20,774	(100.0)

^†^ *Anopheles* spp.: Includes *An. sinensis, An. lesteri, An. lindesai, An. pullus, An. sineroides, An. belenrae, An. kleini*.

**Table 4:** Total number of female mosquitoes collected at mosquito collecting cities with four traps during May to October in 2012.

**Cities**	**Name of traps**	**No. trap**	**Mosquitoes**	**No. species**	**Trap nights**	**TI^†^**
Cheongju	BL	4	250	9	63	10.5
	BG	4	1,821	13	455	75.8
	FP	4	1,913	12	478	79.7
	GV	4	41	5	10	1.7
Nonsan	BL	4	1,352	9	338	56.3
	BG	4	972	11	243	40.5
	FP	4	801	9	200	33.3
	GV	4	59	6	15	2.5
Ansan	BL	3	65	9	22	3.7
	BG	3	6,075	15	2,025	337.5
	FP	3	3,363	12	1,121	186.8
	GV	3	47	5	16	2.7
Yeoju	BL	4	96	5	24	4.0
	BG	4	957	10	239	39.8
	FP	4	1,125	11	281	46.8
	GV	4	31	6	8	1.3
Hwachon	BL	4	1,130	7	283	47.2
	BG	4	306	8	77	12.8
	FP	4	349	9	87	14.5
	GV	4	21	6	5	0.8
Total		76	20,774	20	273	45.5

^†^TI (Trap Index): Average number of mosquitoes per trap night.

BL: Black light trap, BG: BG sentinel trap, FP: Fay prince trap, GV: Gravid trap.

### Virus detection and phylogenetic analysis

Of the 933 pools of mosquitoes tested by real-time RT-PCR, 6 JEVs and 2 chaoyang viruses were detected from 2 species of *Culex* and *Aedes vexans* mosquitoes, respectively (Table 5). In detail, JEVs were detected in *Culex orientalis* and *Culex pipiens* mosquitoes caught in mid August to early September in Hwacheon, Ansan, and Yeoju cities (Table 6). The JEV-positive mosquitoes were caught at all the four habitats (cowshed, downtown area, forest, and swamp) using three collecting traps except the Gravid trap. It is notable that the RT-PCR protocols used in this study allowed the detection of JEV harbored in only one of five mosquitoes (Table 6). The chaoyang virus was detected in the *Aedes vexans* mosquito, which is the dominant species throughout the country. We did not further analyze this virus because it was outside the scope of this study.

**Table 5:** Results of flavivirus detection from field-caught mosquitoes.

**Species**	**Total (%)**		Tested pool*	**JEV**	**Other Flavivirus**
*Culex bitaeniorhynchus*	50	(0.2)	16	0	0
*Culex hayshii*	4	(0.0)	2	0	0
*Culex inatomii*	470	(2.3)	16	0	0
*Culex mimeticus*	1	(0.0)	1	0	0
*Culex orientalis*	498	(2.4)	83	5	0
*Culex pipiens*	9,295	(44.7)	264	1	0
*Culex rubensis*	1	(0.0)	1	0	0
*Culex tritaeniorhyncus*	10	(0.0)	7	0	0
*Culex vagans*	5	(0.0)	2	0	0
*Aedes albopictus*	564	(2.7)	64	0	0
*Aedes vexans*	3,744	(18.0)	168		2^‡^
*Ochlerotatus dorsalis*	6	(0.0)	4	0	0
*Ochlerotatus koreicus*	625	(3.0)	70	0	0
*Ochlerotatus nipponicus*	2	(0.0)	NT	-	-
*Anopheles* spp.^†^	2,045	(9.8)	NT	-	-
*Armigeres subalbatus*	1,132	(5.4)	145	0	0
*Coquillettidia ochracea*	115	(0.6)	14	0	0
*Culiseta bergrothi*	1	(0.0)	1	0	0
*Mansonia uniformis*	2,176	(10.5)	66	0	0
*Tripteroides bambusa*	30	(0.1)	9	0	0
Total	20,774		933	6	2

^†^ *Anopheles* spp.: Includes *An. sinensis, An. lesteri, An. lindesai, An. pullus, An. sineroides, An. belenrae, An. kleini*; *An. sinensis* was not used in the virus survey.

*Tested pools: ≤50/1pool, separated by locality and time.

^‡^ Identified as Chaoyang virus by sequencing analysis and NCBI-BLAST search (data not shown).

NT: Not tested.

**Table 6:** Japanese encephalitis viruses detected from mosquitoes in this study.

**Code**	**Mosquito species (numbers/pool)**	**Collection date**	**Location**	**Trap^†^**	**GenBank Accession no.^‡^**
K12HC959	*Culex orientalis* (12)	2012-08-16	Hwacheon (swamp)	BL	KJ420589
K12AS1148	*Culex pipiens* (50)	2012-08-29	Ansan (swamp)	BG	KJ420590
K12AS1151	*Culex orientalis* (5)	2012-08-29	Ansan (swamp)	BG	KJ420591
K12YJ1174	*Culex orientalis* (46)	2012-09-06	Yeoju (cowshed)	FP	KJ420593
K12YJ1182	*Culex orientalis* (30)	2012-09-06	Yeoju (forest)	BG	KJ420594
K12YJ1203	*Culex orientalis* (1)	2012-09-06	Yeoju (downtown)	BG	KJ420592

^†^ BL: CDC Black-Light trap, BG: BG Sentinel trap, FP: Fay-Prince trap

^‡^ K12YJ1174 and K12YJ1182 are partial length of envelope gene. Others are complete length of 1,500 nts.

**Figure 2:**
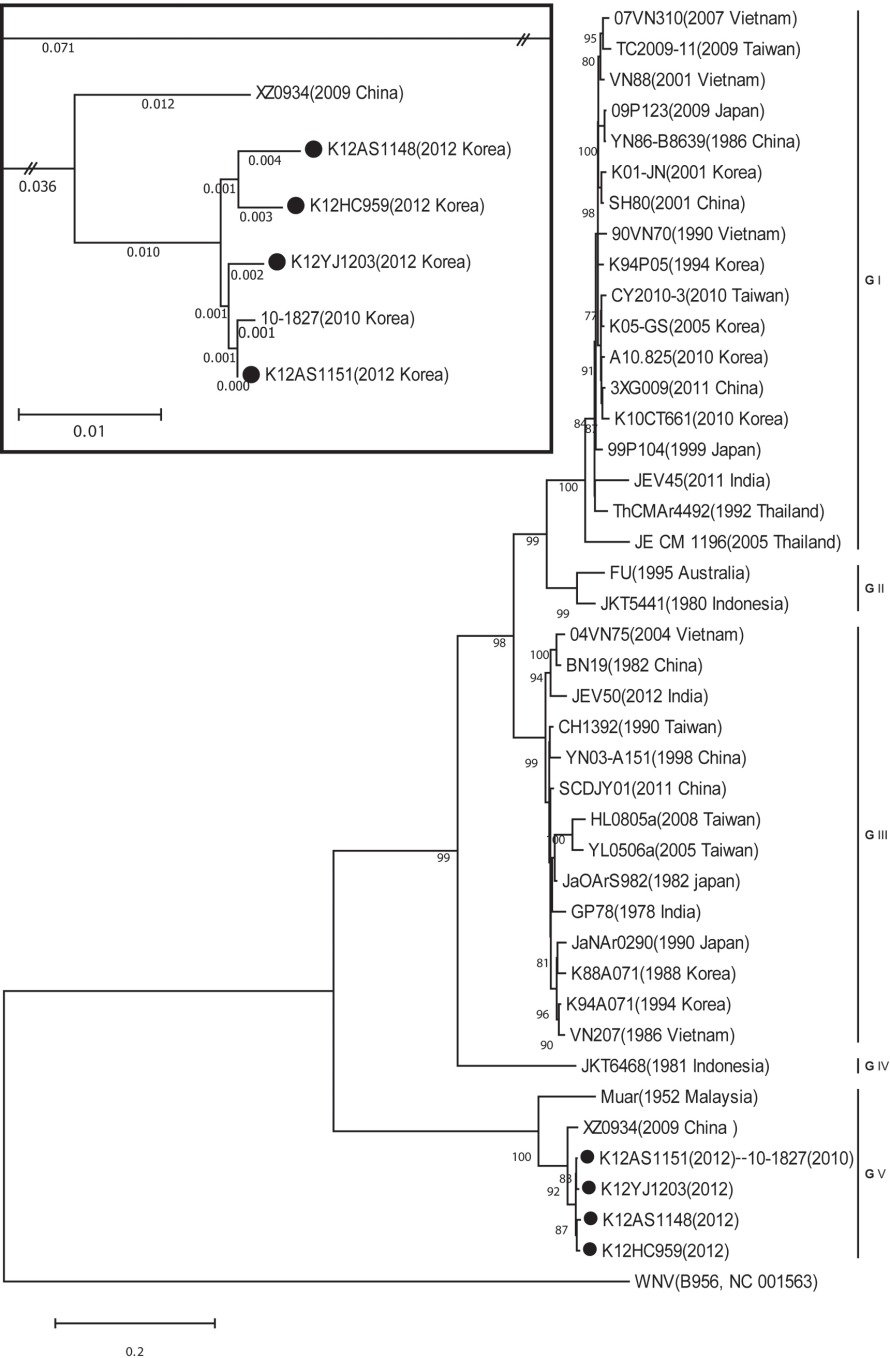
Phylogenetic analysis of Japanese encephalitis virus (JEV) based on the complete envelope gene (1,500 nt) from 42 strains representing each genotypes and countries. The Maximum-Likelihood tree was constructed with a substitution model of Tamura-Nei plus gamma distribution using MEGA software 6.06. West Nile virus (WNV, B956 strain, NC_001563) was used as the outgroup in the tree. Branch reliability is indicated by the percentage of bootstrap values at each node (1,000 replications). The scale bar indicates the number of base substitutions per site. JEVs detected in this study were marked with a closed circle. The left side of the tree is omitted for ease of understanding. In a small rectangle, the topology of genotype V sequences in the original tree is presented. The branch length between K12AS 1154 and its ancestral node is zero and is edited to make the ancestor-descendant relationship clear.

The nucleotide sequences of six JEVs showed sequence similarity ranging from 99.5% to 100% based on the NS 5 gene (180–200 nucleotides). When an NCBI-BLAST search was done for each sequence, the highest hit was the sequence of JEV genotype V. To confirm the genotype, we amplified five fragments covering the complete envelope gene using genotype specific primers. We named the resultant sequences according to the country, year, location and sample number. For example, in the sequence named K12HC959, K means Korea, 12 is a two-digit marking of the sampling year, HC is the abbreviation of location and 959 is the sample number. Among the six JEVs, four pools were successful in having their complete envelope gene sequenced. Only partial sequences around 490 nucleotides were obtained for K12YJ1174 and K12YJ1182 (Table 6). Instead of the two partial sequences, we used K12YJ1203 as a representative sequence which was from the same mosquito species, but acquired on 6 September in Yeoju. An NCBI-BLAST search showed all the envelope gene sequences were best matched to those of JEV genotype V. When phylogenetic analyses were performed with other JEV strains representing each genotype and geography, Korean JEVs were able to be divided into genotypes I, III, and V on the NJ tree (Fig. 2). Both the NJ and ML trees showed the same genotype classification, albeit with differences in branch lengths and bootstrap values. All the JEVs detected in this study were grouped into genotype V, together with the findings of Muar (1952, Malaysia), XZ0934 (2009, China) and 10–1827(2010, Korea). We edited the original tree to make the relationship between the Korean JEV genotype V sequences clear. Because branch length from K12AS1151to its ancestral node was zero in the original tree, we moved the descendant node (K12AS1151 and 10–18927) back to its ancestral node (Fig. 2). Genotype changes in Korean JEVs were shown in 1994 (genotype III → I) and in 2010 (genotype I → V) in the tree. Four JEV sequences obtained in this study showed a nucleotide sequence similarity of 99.1%–99.7% and an amino acid sequence similarity of 99.2%–100% (Table 7). When all seven genotype V sequences in the world were compared to each other, the sequence similarity was 89.9%–99.9% and 95.2%–100% at the nucleotide and amino acid sequence levels, respectively. The genotype V group showed mean sequence divergence ranging from 21.9%–22.8% against the other four genotypes, while those genotypes showed mean sequence divergence of 10.8–18.2% each other.

**Table 7:** Nucleotide sequence similarity and divergence of envelope gene among JEV genotype V.

	**Muar**	**XZ0934**	**10–1827**	**K12AS1148**	**K12AS1151**	**K12HC959**	**K12YJ1203**
Muar		90.2	89.9	90.1	90.1	89.9	89.9
XZ0934	9.8		97.3	97.0	97.5	97.1	97.3
10–1827	10.1	2.7		99.1	99.9	99.3	99.5
K12AS1148	9.9	3.0	0.9		99.3	99.2	99.1
K12AS1151	9.9	2.5	0.1	0.7		99.4	99.7
K12HC959	10.1	2.9	0.7	0.8	0.6		99.2
K12YJ1203	10.1	2.7	0.5	0.9	0.3	0.8	

The upper right of diagonal shows percentage sequence similarity and the lower left of diagonal shows sequence divergence (p-distance).

## Discussion

In this study we detected six JEVs from *Culex orientalis* and *Culex pipiens* mosquitoes. These results may provide answers to the question of why JE patients have increased abruptly since 2010 and why the outbreaks have occurred in an unexpected province where the density of *Culex tritaeniorhynchus* mosquitoes was very low throughout the JE season. Recently, two research groups in Italy and Korea have reported that *Culex pipiens* mosquitoes may transmit JEV [13,14]. In addition, JEV was isolated from *Culex pipiens* which were collected during winter in Korea in the early 1970s. Despite the low vector competence that *Culex pipiens* displays in laboratory studies [15], our study strongly supports the previous finding that this mosquito is infected by JEV, and may transmit the virus.

Here, the name *Culex pipiens* is used to represent *Culex pipiens pallens* Coquillett. In Korea, the *Culex pipiens* complex consists of two species of mosquitoes, *Culex pipiens pallens* and *Culex pipiens molestus* Forskal. However, it is agreed that *molestus* is not a subspecies, but rather a strain of *Culex pipiens pallens* [16].

This is the first report of JEV detection in a *Culex orientalis* mosquito that was distributed in Far-East Asia, including China, Japan, Korea, Siberia, and Taiwan. However, this mosquito is not likely to cause a large impact JE outbreak because of its bionomics. The larvae of this species live strictly in fresh water, such as slowly moving ponds and streams, on mountains in Korea, and the adults apparently do not feed on humans [17]. However, *Culex pipiens* is the most common mosquito in human dwelling areas in Korea. The larvae of this mosquito occur in a very wide variety of artificial containers, or other types of stagnant water, such as ditches, gutters, ground pools, etc. They prefer polluted water containing abundant organic matter [17]. The adult mosquito is commonly considered to be an avian blood feeder, but a recent study has shown that the mammal feeding rate was comparatively high [18]. Therefore, the role of *Culex pipiens* and *Culex orientalis* in the transmission of JEV should be studied urgently.

In this study virus isolation was not attempted because dead mosquitoes were stored for several weeks before the RT-PCR. According to our long-term experience, and another paper [19], virus recovery could not be expected in such a circumstance. Instead, we sequenced the complete envelope gene using genotype specific primers. Interestingly, all the JEVs detected in this study belonged to genotype V, which was the second report in Korea to do so, after a US military research group detected it in 2010 in north Gyeonggi-do province [7]. Historically, genotype III was the dominant strain worldwide until the latter part of the 20th century. Then, a JEV genotype shift from type III to I was reported in many regions, and genotype I became recognized as the dominant strain in many countries [20]. Genotype V, on the other hand, is a very rare strain that was first reported from an encephalitis patient in Malaysia in 1952 [8] and then in 2009 in China [9]. In Korea, JEV genotype III strains were also dominant until the genotype I was first isolated in 1994. Since then, only genotype I strains were isolated until 2010 [21]. The pathogenic properties of the genotype V strain have only rarely been documented, and there is no relevant information on whether current genotype III-based vaccines could protect against this new genotype. Thus, we could not determine the effects of the emergence of this new genotype on the current JE outbreak in South Korea.

JEV vector surveillance is one of the most important tools for providing information on the distribution, intensity, and abundance of circulating viruses that can be used to create strategies for public health [14]. As a mosquito collection tool, we traditionally used a black-light trap in cowsheds or downtown areas. However, this trap attracts only some of the dominant species of mosquitoes, and consequently failed to collect the full diversity of species distributed in the natural ecosystem. Therefore, we tried four different trapping methods in various habitats. The number of collected mosquito species has showed a strong contrast by trap type (Table 4). This strategy proved to be a success in that JEV-positive pools were collected in both forests and swamps as well as the traditional sampling sites, and four out of six positive pools were collected with a BG sentinel trap. To accurately understand the circulation of viruses in nature, we recommend the collection of mosquitoes in various habitats.

In conclusion, we found that two new *Culex* mosquitoes, *Culex orientalis* and *Culex pipiens* may transmit JEVs as a secondary or regional vector. Additionally, we confirmed that JEV genotype V strains were disseminated in at least the Gangwon-do and Gyeonggi-do provinces. These findings broaden our knowledge on the vector diversity of JEV and highlight the need to review the current vector surveillance protocol in Korea.
